# Low magnitude high frequency vibration promotes adipogenic differentiation of bone marrow stem cells via P38 MAPK signal

**DOI:** 10.1371/journal.pone.0172954

**Published:** 2017-03-02

**Authors:** Qian Zhao, Yuezhi Lu, Haiyang Yu, Xueqi Gan

**Affiliations:** State Key Laboratory of Oral Diseases, West China Hospital of Stomatology, Sichuan University, Chengdu, China; University of Sheffield, UNITED KINGDOM

## Abstract

Low magnitude high frequency vibration (LMHFV) has been mainly reported for its influence on the musculoskeletal system, particularly the bone tissue. In the bone structure, osteogenic activity is the main focus of study with regards to LMHFV. However, adipogenesis, another important mode of differentiation in the bone marrow cavity that might be affected by LMHFV, is much less researched. Furthermore, the molecular mechanism of how LMHFV influences adipogenesis still needs to be understood. Here, we tested the effect of LMHFV (0.3g, 40 Hz, amplitude: 50μm), 15min/d, on multipotent stem cells (MSCs), which are the common progenitors of osteogenic, chondrogenic, adipogenic and myogenic cells. It is previously shown that LMHFV promotes osteogenesis of MSCs. In this study, we further revealed its effect on adipo-differentiation of bone marrow stem cells (BMSCs) and studied the underlying signaling pathway. We found that when treated with LMHFV, the cells showed a higher expression of PPARγ, C/EBPα, adiponectin and showed more oil droplets. After vibration, the protein expression of PPARγ increased, and the phosphorylation of p38 MAPK was enhanced. After treating cells with SB203580, a specific p38 inhibitor, both the protein level of PPARγ illustrated by immunofluorescent staining and the oil droplets number, were decreased. Altogether, this indicates that p38 MAPK is activated during adipogenesis of BMSCs, and this is promoted by LMHFV. Our results demonstrating that specific parameters of LMHFV promotes adipogenesis of MSCs and enhances osteogenesis, highlights an unbeneficial side effect of vibration therapy used for preventing obesity and osteoporosis.

## Introduction

Low magnitude high frequency vibration (LMHFV) is defined as LM < 1×g, (1g = 9.8m/s^2^)and HF = 20–90 Hz[1]. It can be applied by placing a subject on a vibrating platform or vibrating cells seeded in culture plates or 3D structures[2]. LMHFV is becoming a potential approach to repair and regenerate the musculoskeletal system[2], and lose weight[3]. Most in vivo and in vitro trials have shown that LMHFV exerts anabolic effects on skeletal metabolism by promoting osteogenic activity[1,4–6]. In the marrow cavity, there is a balance between bone and adipose tissue[7]. New strategies that are being designed to prevent osteoporosis involve regulating the fate of the progenitor, mesenchymal stem cells (MSCs), which are capable of differentiation into osteoblasts, adipocytes, fibroblasts, chondrocytes, and myocytes[8]. Therefore, if LMHFV could bias the fate of MSCs in the bone marrow cavity to osteogenic lineage, it would be beneficial for the bon etissue. Vibration promoting osteogenesis of MSCs has been studied: research has shown that MSC under "nanokicking" (1kHZ vibration), can achieve osteogenesis[9]; the application of low intensity pulsed ultrasound can restore osteogenesis in Ad-hMSCs[10]. However, the effect of vibration on adipogenesis in the bone marrow cavity within the body is much less researched. Since the LMHFV (0.3g, 40Hz) has been shown to promote MSCs differentiating to osteogenic lineage[1, 11], in our work, we wanted to test how this vibration affected adipogenic differentiation of BMSCs. If the adipogenesis were inhibited, it would support the application of LMHFV in clinical therapies more reasonably and safely.

p38 MAPK along with extracellular signal-regulated kinases (ERK), Jun N-terminal kinases (JNK) and ERK5 constitute the mitogen activated protein kinases (MAPKs), which are evolutionarily conserved enzymes that trigger a wide range of cellular responses to deal with extracellular stimuli. MAPKs are signal components that are characterized by a core triple kinase cascade. Initially, an upstream signaling protein activates a MAP kinase kinase kinase(MAPKKK), which then phosphorylates a MAP kinase kinase (MAPKK). And the activated MAPKK phosphorylates the third layer of the cascade, MAPK[12]. P38 can be activated by extracellular stimuli such as UV light, inflammatory cytokines, heat, osmotic shock and growth factors, thus playing key roles in inflammation, apoptosis, cardiomyocytes hypertrophy, senescence and tumor suppression, as well as in development and differentiation[13]. Many studies describe the role of p38 MAPK ina dipogenesisof 3T3-L1 preadipocyte cell lines and MSCs. After treatment with a highly specific p38 inhibitor SB203580, 3T3-L1 cells expressed lesser C/EBPδ than those that were non-treated[14]. During initial differentiation of 3T3-L1preadipocytes, p38 MAPK was activated, and the adipogenesis was effectively blocked by using SB203580[15]. The mRNA expression of adipogenic genes of mouse BMSCs were downregulated when phosphorylation of p38 MAPK was increased[16]. Mechanical forces have also been reported to activate the p38 pathway. Compressive force apparently augmented the expression of Runx2[17] and chondrocyte-specific genes[18] by inducing the phosphorylation of p38 MAPK. Stretching force(18% strain) increased the phosphorylation of p38 in human aortic smooth muscle cells[19]. When MSCs were subjected to combined treatment of vibration and connective tissue growth factor, p38 phosphorylation was significantly suppressed[20].

Based on the two aspects above, previous studies have shown a close interconnection between adipogenesis, p38 MAPK and mechanical stimuli including vibration. Thus, our hypothesis tested if LMHFV could modulate adipo-differentiation of BMSCs through p38 MAPK pathway.

## Materials and methods

### Primary culture of rat BMSCs

**Fig 1:**
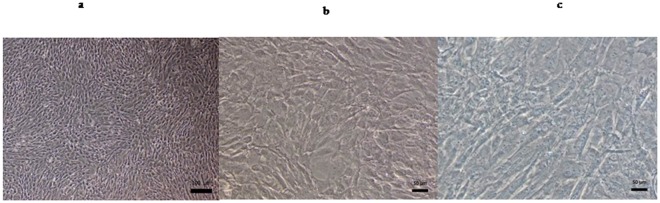
Passage3 of BMSCs from Sprague Dawley rat under microscope(Olympus, Japan). Representative images:(**a**)scale bar = 100 μm,(**b**)scale bar = 50 μm, (**c**).scale bar = 50 μm.

Sprague–Dawley rats from Sichuan University West China Animal Laboratory Center were sacrificed by dislocating the spine. The BMSCs were acquired from posterior tibias and femurs. This research was granted permission by State Key Laboratory of Oral Disease ethics committee. The bone marrow cells were flushed out from the marrow cavity of posterior tibia and femur of 3-week-old male Sprague–Dawley rats, in a culture medium consisting of DMEM (Gibco, Grand Island, NY), 10% FBS (Gibco, Grand Island, NY) and 1% penicillin-streptomycin. The primary cells were cultured in a humid environment of 37°C and 5% CO_2_. Non-adherent cells were discarded by changing culture medium 24 h later, and subsequently, until the cells reached 80–90% confluent, the culture medium was replaced every 2 days. 0.25% trypsin (Gibco BRL) was applied to detach the cells, then subculture to passage two or three [1]. When the BMSCs were 100% confluent(Fig 1a–1c), they were used for the rest of the experiments.

### Adipogenic induction

We used OriCell^™^ SD Rat Mesenchymal Stem Cell Adipogenic Differentiation Medium (Cyagen Biosciences, Guangzhou, China). The BMSCs were transferred to adipogenic induction medium A (1ul/ml dexamethasone, 1ul/ml rosiglitazone, and 1ul/ml isobutyl-methylxanthine and 2ul/ml insulin), with a density of 1x10^6^/ml. After 72 h, the medium A was replaced by adipogenic induction medium B (2ul/ml insulin) for 24 h. Hence, the total induction cycle was for96 h. In our work, 1 to 3 cycles were operated.

### LMHFV application

**Fig 2:**
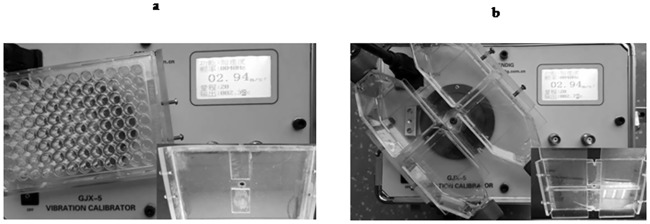
(a)Vibration sensor with fixator for culture plates, and(b) vibration sensor with fixator for culture flasks.

Two types of fixators were designed for the culture flasks and plates, respectively. The fixator can be mounted on the platform of the vibration sensor (Beijing Sending Technology, Beijing, China; Fig 2a and 2b). After the fixator was installed horizontally, we placed the flasks or plates with BMSCs tightly on the fixator by screws. Then, the vibration (magnitude: 0.3×g, frequency: 40 Hz, amplitude: 50μm) was transmitted to cells for 15 min each day for a period of 1, 4, 8 and 14 days. Vibration and induction were carried out in the same period.

### Oil red O staining

Cells after induced for 8 days, were seeded on 6-well plates (1×10^6^/ml). After adherence, the cells were fixed in 10% paraformaldehyde for 20 min, washed in PBS, stained with oil red o solution (Cyagen Biosciences, Guangzhou, China) for 20 min, soaked in 60% isopropanol and then washed with PBS. Images were captured by bright field microscope (Olympus, Tokyo, Japan) and analyzed by software Image J (National Institute of Health, Maryland, USA).

### Quantitative real-time RT-PCR

Total mRNA was extracted from specimens on day 1, 4, 8 and 14 by adding 1ml trizol (TAKARA, Dalian, China), 200μl chloroform and 500μlisopropanol, successively. Value of A(260) /A(280) was calculated to confirm the purity of the total RNA. Synthesis of cDNA was performed using PrimeScript RT reagent kit with gDNA Eraser (Perfect Real Time) (Takara Biotechnology, Dalian, China). Primers list is given in Table 1. The amplification reaction was run in the ABI PRI SM 7300 Sequence Detection system (Applied Biosystems, Foster City, CA, USA) using SYBR Premix Ex Taq^™^II(TliRNaseH Plus) Kit (Takara Biotechnology, Dalian, China). The program was set to 3 steps with denaturation at 95°C for 30 s, annealing at 95°C for 5 s and elongation at 60°C for 31 s. Specificity of the PCR products were examined by melting curve analyses. House-keeping gene GAPDH was used to normalize the values of relative gene expression[21].

**Table 1:** Primers used for real-time RT-PCR.

Gene	Primer sequence	GeneBank accession
	The house-keeping gene	
GAPDH	F: TATGACTCTACCCACGGCAAGT	NM_002046
	R: ATACTCAGCACCAGCATCACC	
	Adipogenic marker genes	
PPARγ	F: 5' TTATGGAGCCTAAGTTTGAGTTTGC 3'	NM_001145366
	R: 5' TTGTCTTGGATGTCCTCGATGG 3'	
CEBPα	F: 5' GAAGTCGGTGGATAAGAACAGCA 3'	NM_001287577
	R: 5' CTCCAACACCTTCTGCTGCGT 3'	
Adiponectin	F: 5' GTCCCTCCACCCAAGGAAACT 3'	NM_144744
	R: 5' CTCCTGTCATTCCAGCATCTCC 3'	

List of primers used in the study for gene expression analysis.

### Western blot

PPARγ, p38, phospho-p38 protein in BMSCs under different culture conditions were measured. Proteins were harvested with whole cell lysis assay kit (Keygen, Nanjing, China). Cells were washed in cold PBS, followed by addition of 1ml lysis buffer, then agitated strongly for 4 seconds at 4°C, and placed on the ice for 4 min. This was repeated five times. Cell lysates acquired were centrifuged at 12,000 g for 5 min at 4°C, then the supernatant protein was harvested and total protein concentration was determined using BCA protein assay kit (Keygen, Nanjing, China). After separating the protein samples on 10% SDS-PAGE gels, transferring to polyvinylidenedifluoride membranes (Millipore, USA), these membranes were immunoblotted by rabbit anti-PPARγ polyclonal antibody (Abcam, Cambridge, UK)) at 4°C overnight and incubated with secondary antibody (Abcam, Cambridge, UK)) at room temperature for 1h. PPARγ, p38 and phospho-p38 were visualized using enhanced chemiluminescence reagents (Millipore, USA). The immunoblots were quantified with software Image J (National Institute of Health, Maryland, USA).

### Application of SB203580

The BMSCs in adipo-induction medium without LMHFV were defined as control group. BMSCs subjected to LMHFV (0.3×g, 40Hz) and adipogenic medium were sorted as vibration group. To confirm the involvement of p38 in the adipogenesis of BMSCs, SB203580 (Byotime, Shanghai, China), a highly selective inhibitor of p38 [14,15,18]was used. The inhibitor was dissolved in dimethyl sulphoxide (DMSO; Sigma). BMSCs were placed in adipogenic medium with 10uM SB203580 for 2h [18]before exposure to LMHFV. The inhibitor was replenished together with the medium in the following days. The untreated cells were cultured in the induction medium in the same amount of DMSO (0.099%) only.

### Immunofluorescence (IF) staining

Cells with or without LMHFV application and those with or without SB203580, were seeded on millicell EZ 8-well slide. After adherence, they were washed in PBS, fixed in 4% buffered (0.01mol/L PBS, pH 7.2, 0.1%DEPC) paraformaldehyde for 30 min at room temperature, and blocked by 0.5% bovine serum albumin (BSA) for 15 min. After overnight incubation with rabbit anti-PPARγ polyclonal antibody (1:60) (Abcam, Cambridge, UK)[15] at 4°C, cells were subsequently incubated with secondary antibody conjugated to Rhodamine (Hebei Bio-high Technology Deve co., Hebei, China) for 20 min at 37°C. After rinsing in PBS, cells were observed under the Olympus IX 71 inversed fluorescent microscope (Olympus, Tokyo, Japan). Images were captured by Image-Pro Plus version 6.0 (Media Cybernetics) and analyzed by Image J software (National Institute of Health, Maryland, USA).

### Statistical analysis

The data were presented as mean±standard deviation and analyzed using one-way ANOVA by software SPSS, version 17.0 (SPSS Inc., Chicago, IL, USA). Statistically significant values were considered at *P*<0.05. Three independent experiments were performed and all assays were repeated twice.

## Results

### LMHFV increased the formation of lipid droplets during the adipogenic process of BMSCs

**Fig 3:**
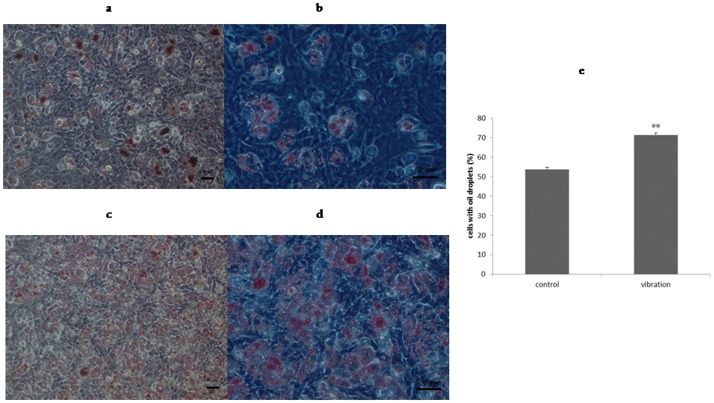
LMHFV increased the formation of lipid droplets during the adipogenic process of BMSCs. BMSCs after induction for 8 days. Staining is done by oil red o staining: control group cells (**a**)scale bar = 50 μm, (**b**)scale bar = 50 μm; vibration group cells(**c**) scale bar = 50 μm, (**d**)scale bar = 50 μm; (**e**) quantification of cells with oil droplets increased by vibration, *P*<0.01(**).

After being cultured in adipogenic medium for 8 days, cells were stained by oil red o (Fig 3). The control group showed large numbers of small oil droplets (Fig 3a and 3b). However, in vibration group, cells with lipid droplets were much more common, and their percentage increased from 53.75% to 71.43% upon vibration (Fig 3c–3e; *P*<0.01). This suggested that LMHFV could significantly promote differentiation of adipogenesis, increasing the number of mature adipocytes.

### mRNA expression of adipogenic genes were strengthened by LMHFV

**Fig 4:**
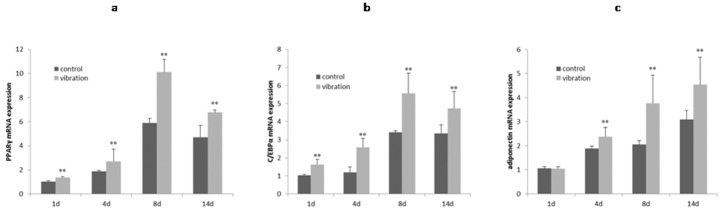
mRNA expression of adipogenic genes were strengthened by LMHFV. Effects of LMHFV on adipogenic marker genes: when cells exposed to LMHFV, mRNA expression of (**a**)PPARγ, (**b**)C/EBPα and (**c**)adiponectin, were higher; *P*<0.01(**).

Quantitative real-time RT-PCR results indicated the same trend of mRNA expression for PPARγ and C/EBPα. On day 1, 4, 8 and 14, the mRNA expression of PPARγ and C/EBPα apparently increased after vibration, and showed the peak value on day 8(Fig 4a and 4b; *P*<0.01). Similarly, after exposure to vibration, mRNA level of adiponectin increased significantly on day 4, 8 and 14, with the highest expression on day 14(Fig 4c; *P* < 0.01), while showing no differences on day 1.

### Expression of adipogenesis related protein was promoted by LMHFV

**Fig 5:**
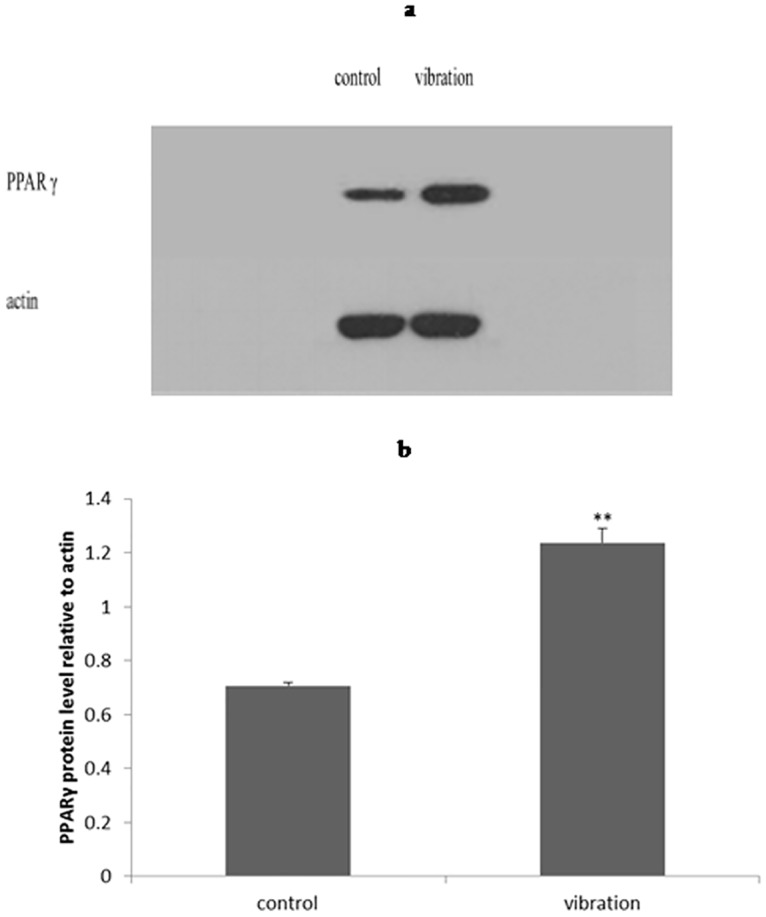
Western blot on day 8 indicated PPAR γ protein expression was enhanced under vibration: representative gel of PPAR γ (a) and quantification of PPARγ protein level (b); *P*<0.01(**).

We used PPARγ to represent an adipogenesis related protein. Western blot on day 8 revealed that PPARγ greatly increased under LMHFV from 0.71 to 1.24(*P*<0.01)(Fig 5a and 5b), which meant adipogenesis of BMSCs was boosted by vibration.

### LMHFV activated p38 MAPK

**Fig 6:**
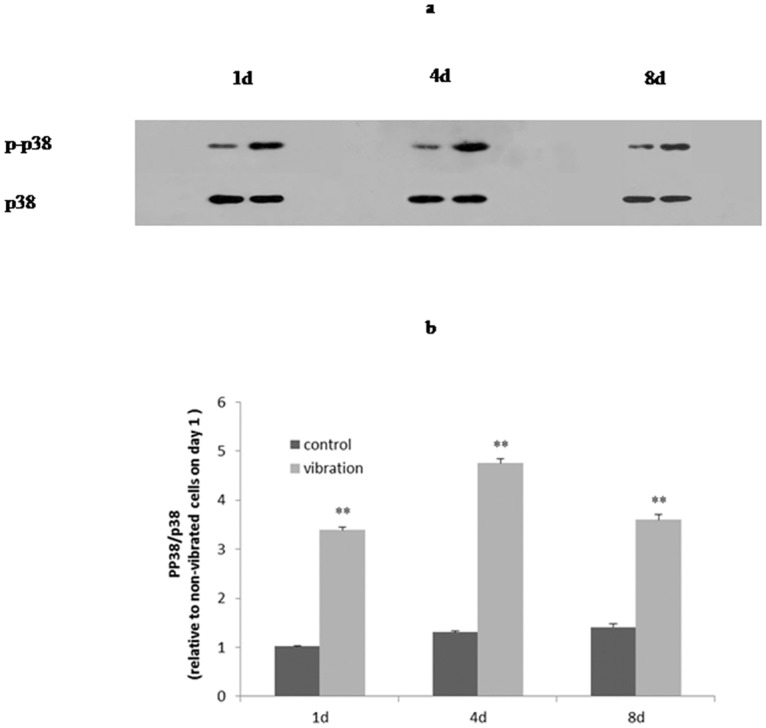
LMHFV activated p38 MAPK. P-p38/p38 rose up by LMHFV on day 1, 4 and day 8:representative gel of p-p38 and p38 (a), and quantification of p-p38 protein level (b);*P*<0.01(**).

The results of western blot on day 1, 4 and 8 indicated phospho-p38 were upregulated under LMHFV (Fig 6a and 6b; *P*<0.01). Sincephospho-p38 representsactivep38, the upregulation of phospho-p38 implied p38 was activated by vibration.

### Activated p38 MAPK increased adipogenesis of BMSCs

**Fig 7:**
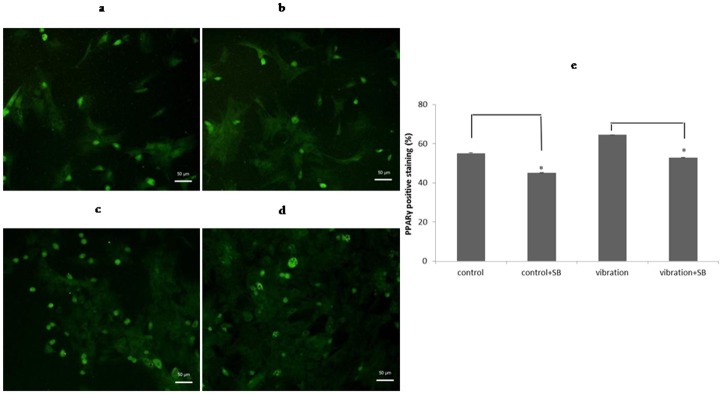
Activated p38 MAPK increased adipogenic gene expression of BMSCs. Protein level of PPAR γ on day 8 was detected by immunofluorescence staining (scale bar = 50 μm): (**a**)control and (**b**)control+SB; (**c**) vibration and(**d**)vibration+SB;(**e**)after inhibition, PPAR γ positive staining decreased, *P*<0.05(*).

**Fig 8:**
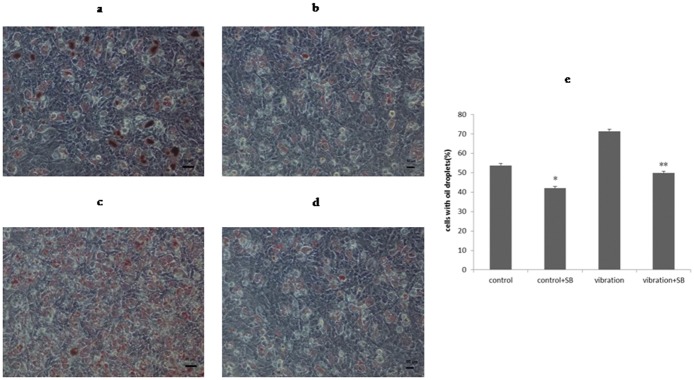
Activated p38 MAPK increased adipocytes formation of BMSCs. Cells with oil droplets on day 8, were shown by oil red o staining(scale bar = 50 μm): (**a**)control and(**b**)control+SB;(**c**) vibration and(**d**)vibration+SB;(**e**)after inhibition, cells with oil droplets decreased, *P*<0.05(*),*P*<0.01(**).

The protein expression of PPARγ and oil droplet numbers on day 8 were used to represent the level of adipogenic differentiation, and were assessed by IF staining and oil red o staining, respectively. With or without vibration, application of SB203580 suppressed PPARγ expression (Fig 7a–7e). PPAR γ positive staining decreased from 55.17% to 45.13% in control related groups; from 64.48% to 52.87% in vibration related groups, respectively (Fig 7e; *P*<0.05). Also, as expected, the formation of lipid droplets was decreased (Fig 8a–8e); after inhibition, cells with oil droplets in control group decreased from 53.75% to 41.99%, and those in vibartion group decreased from 71.43% to 49.83% (Fig 8e; *P*<0.01). Altogether, this implied that p38 MAPK was positively involved in the progress of adipo-differentiation of BMSCs.

## Discussion

In our work, we studied the effect of LMHFV (magnitude: 0.3×g, frequency: 40 Hz, amplitude: 50μm) on BMSCs adipo-differentiation, from the modulation of cellular and molecular levels. When vibration was applied, the cells were cultured in adipogenic induction medium. We showed that LMHFV induced BMSCs to express higher adipogenic mRNA and protein levels, and the active p38 played a positive role during the progress of LMHFV-induced adipogenesis.

PPARγ is well established as a prime regulator of differentiating adipogenic cells [22]. C/EBPα is a key factor of adipogenesis, having an important influence on the regulation of adipocyte differentiation[23], and adiponectin is one of the adipocyte predominant proteins. These three adipogenic genes showed peak expression in different time periods in our study. On day 1, C/EBPα showed the highest gene expression; PPARγ was highly expressed on day 8; and the expression of adiponectin kept rising from day 1 until day 14 to the highest level, which suggested that these genes affected adipogenesis in their respective dominant period. After vibration, more cells showed oil droplets in their cytoplasm, implying production of more mature adipocytes [24]. These results suggested that the LMHFV we tested could increase the commitment of BMSCs to adipogenesis.

There might be two explanations for this. Firstly, MSCs were in different environments. The key environment is of significance for lineage selection of MSCs[8]. Previous studies which demonstrated a certain vibration strengthened osteogenesis while suppressing adipogenesis, were studying this indifferent cellular environments than the environment we studied: C3H10T1/2 mesenchymal stem cells were cultured in normal culture medium with the functional matrix[25]; BMSCs were harvested from male C57BL/6J mice after they were vibrated within the mice body[8]. This implies that the BMSCs were in a much more complex body liquid environment. However, in our study, BMSCs were cultured in adipo-induction medium. In this case, extracellular fluid environment could induce MSCs to differentiation to adipogenic lineage, compared with the osteo-induction or the more complex liquid environment in other studies[1,8,25]. Based on this point, we might conclude the effect of LMHFV on MSCs lags behind that of adipo-induction medium, leading to the initial adipogenic differentiation induced by the culture medium. Then, vibration acted on the pre-adipogenic cell lines to promote further differentiation. This deduction is consistent with the previous evidences[8,26,27]. Secondly, vibration was applied with a different scheme compared to the previous studies. Varying application scheme has been shown to have an important effect in the outcomes of vibration stimuli. Altering the bout or rest period could alter the results[28]. In our study, LMHFV was added to cells 15 minutes each day, the bout was shorter and the interval period was longer, compared with 30 min/12h[1] and 20min/d[11]). In order to verify these explanations, more studies are needed to be performedin the future.

Regarding our hypothesis on p38 MAPK, it has been reported that mechanical stimuli could activate p38 MAPK[17–20],and in our study, phospho-p38 expression was higher after vibration, suggesting that p38 could be activated by LMHFV. Meanwhile, vibrated cells expressing more PPARγ protein and showing more oil droplets than those non-vibrated, revealed vibration promoted adipo-differentiation. On the other hand, previous evidences have also shown that p38 MAPK could be activated or inactivated during adipogenes is process[29–31]. According to these, we speculated that this promotion of LMHFV was modulated by p38 MAPK. As expected, in our p38 MAPK inhibition study, PPARγ protein expression and oil droplets number decreased after being treated with SB203580, the specific inhibitor of p38 MAPK [14,15,18]. This supported the association of p38 MAPK and its positive role in LMHFV-induced BMSCs adipo-differentiation.

We tested *invitro* LMHFV promoted adipogenesis of BMSCs. However, whether this same effect couldbe duplicated under different experimental conditions *in vitro* and *in vivo*, is unknown. Increase in the number of adipocytes and lipid accumulation are both important factors that induce obesity[24]. Similarly, osteoporosis often occured when adipose tissue in marrow cavity grows and altered the balance between fat and bone[32]. Thus, before LMHFV could be safely used to treat these clinical problems, there is still much work to be done, both *in vitro* and *vivo*.

In conclusion, we find in the environment of adipogenic induction, LMHFV strengthens the adipogenic marker genes expression at the mRNA and protein levels by activating p38 MAPK.

## Supporting information

S1 File Reviewer 1 question2 supporting certificate. (PDF)

S2 File Data. (PDF)
