# Correction: Low magnitude high frequency vibration promotes adipogenic differentiation of bone marrow stem cells via P38 MAPK signal

**DOI:** 10.1371/journal.pone.0189547

**Published:** 2017-12-07

**Authors:** Qian Zhao, Yuezhi Lu, Xueqi Gan, Haiyang Yu

The third and fourth authors are listed out of order. Please view the correct author order, affiliations, and citation here:

Qian Zhao^1☯^, Yuezhi Lu^1☯^, Xueqi Gan^1^, Haiyang Yu^1^

1 State Key Laboratory of Oral Diseases, West China Hospital of Stomatology, Sichuan University, Chengdu, China

☯ These authors contributed equally to this work.

Zhao Q, Lu Y, Gan X, Yu H (2017) Low magnitude high frequency vibration promotes adipogenic differentiation of bone marrow stem cells via P38 MAPK signal. PLoS ONE 12(3): e0172954. https://doi.org/10.1371/journal.pone.0172954
