# Supplementary material for: Low magnitude high frequency vibration promotes adipogenic differentiation of bone marrow stem cells via P38 MAPK signal
Source: PLoS One. 2017 Mar 2;12(3):e0172954. doi: 10.1371/journal.pone.0172954 (PMC5333869; doi:10.1371/journal.pone.0172954)
Supplement: S1 File — (PDF) [file pone.0172954.s001.pdf]

中国航空工业第十区域成都计量站

国防科技工业 5112 二级计量站

Chengdu Tenth Regional Metrology Station of China Aviation Industry

The 5112 Level 2 Metrology Station of The National Defense Science And Technology Industry

校 准 证 书

Calibration Certificate

证书编号：E201614217
Cert No.

委 托 单 位 Applicant	四川大学
地 址 Add	成都
样 品 名 称 Instrument Name	振动器
型 号 规 格 Model/Type	GJX-5
出 厂 编 号 Ex-factory No.	/
制 造 厂/商 Manufacturer	Beijing Sending Technology

批准人(签字) Approved by 查永康

发证单位(专用章) Issued by (Stamp) 中国航空工业第十区域成都计量站 校准专用章

发证日期 Date Issued 2016 年 Year 10 月 Month 31 日 Date

本实验室地址 Add: 四川省成都市新都区三河场蜀龙路成发工业园
Chengfa Industry Park,Shulong Road, Sanhechang,Xindu District,Chengdu,Sichuan,China
联系电话 Tel: （028）89358722 传真 Fax: （028）89358722 邮编 Post Code: 610503

授权与溯源

Authorization and Traceability

本实验室类别： 校准实验室
Laboratory sort Calibration Laboratory

认 可 单 位： 中国合格评定国家认可委员会/国防科技工业实验室认可委员会
Authorization body CNAS/DILAC

认可证书号： CNAS L0009 / DL090
Accreditation certificate

溯源性： 标准溯源至国家测量标准
Traceability The master used are traceable to National Measurement Standard

校准所使用的主要计量器具

Main standards of measurement used in the Calibration

名称<br>Name	出厂编号<br>Ex-factory No.	不确定度或准确度等级或最大允许误差<br>Uncertainty/Accuracy class/ maximum permissible error	证书编号<br>Cert No.	有效期至<br>Valid date to	发证单位<br>Issued by
动态信号分析仪	5325932	A 级	力 C01 字第 2014030026 号	2017/03/06	304 所
中频振动标准套组	2752217+ 2773823	1%	GFJDJL1001603024 13	2017/03/08	304 所

校准所依据技术文件

Reference Documents For The Calibration (Code、Name)

JJG190-97《电动振动试验台》

校准地点、日期、环境条件

Place and date of the calibration and environmental condition

地点： 国防科技工业 5112 二级计量站
Place

接收日期 2016 年 10 月 28 日
Rec. Date Year Month Date

校准日期 2016 年 10 月 31 日
Date Calibrated Year Month Date

温度 21.0 ℃ 相对湿度 53.8 %
Temperature RH

校 准 结 果

Calibration Results

1：空载

振动器设置 40Hz 加速度 2.97m/s^2

测量值 40.0Hz 加速度 3.109 m/s^2 失真度 1.05%

2：加塑料板

振动器设置 40Hz 加速度 2.65m/s^2

测量值 40.0Hz 加速度 2.970 m/s^2 失真度 1.67%

频率示值校准不确定度 0.012% (k=2)

加速度幅值示值校准不确定度 2.0%(k=2)

校准员(签字) Calibrated by

核验员(签字) Checked by

本证书只对该委托件有效，未经本实验室书面批准，不得部分复制。
This certificate is valid only for the customer equipment and cannot be partly copied if not allowed by the Calibration Laboratory on writing.
