# Supplementary material for: Low magnitude high frequency vibration promotes adipogenic differentiation of bone marrow stem cells via P38 MAPK signal
Source: PLoS One. 2017 Mar 2;12(3):e0172954. doi: 10.1371/journal.pone.0172954 (PMC5333869; doi:10.1371/journal.pone.0172954)

control			vibration			control+SB203580			vibration+SB203580		
164246	161245	169891	217688	216490	224146	125387	132871	128697	156663	148466	154062
307200											
0.534655	0.524886	0.553031	0.70862	0.70472	0.729642	0.408161	0.432523	0.418936	0.509971	0.483288	0.501504
	0.537524			0.714327			0.419873	41.9873		0.498254	49.8254
	0.01429			0.013406			0.012208			0.013635	
	control	c+SB20358	vibartion	v+SB203580							
	53.75239	41.9873	71.43273	49.8254							
	1.43	1.22	1.34	1.36							
	p=0		p=0								

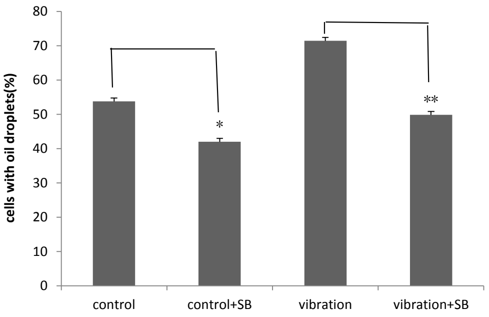

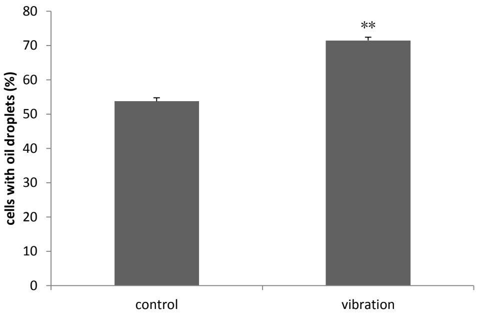

	c1d	v1d	c4d	v4d	c8d	v8d	c14d	v14d	c+14d	v+14d
内参GAPGH										
	20.8545	21.8455	23.1955	22.4522	20.4596	20.4511			17.5422	17.4877
	21.2144	22.4218	23.5677	22.6951	20.8561	20.2189			17.4955	17.6522
	21.0025	21.77366	23.2419	22.2655	20.5322	20.795			17.1455	17.9853
	20.64131	22.5962	23.2012	22.1862	20.0417	20.4936			19.7962	18.7061
	20.73682	21.67337	23.5482	22.4264	21.0785	20.7888			16.7548	19.4959
	21.2145	22.39552	23.1217	22.8221	21.2007	20.7342			20.0016	18.5425
	20.95925	21.97215	23.3598	22.1884	20.5663	20.808			15.825	16.3241
	20.77355	22.18073	23.702	22.1956	20.9371	20.6119			16.3722	17.6124
	21.11196	22.39256	23.3133	22.99248	20.3938	20.7897			16.4107	17.0372
PPAR										
	29.93102	30.39148	31.41559	30.02231	26.89318	26.27334			24.51042	23.79701
	29.32885	30.23846	30.71024	29.42688	26.3563	25.18324			23.54423	23.05337
	29.47082	29.74878	30.96004	29.36012	26.50026	25.7376			23.18118	23.69131
	29.79476	31.34417	31.17968	29.34462	26.26203	26.20201			26.74534	25.1444
	28.88171	29.35748	30.55681	29.45895	27.35621	25.3561			22.60061	24.89621
	29.66153	30.37	31.26386	29.69713	27.52915	26.02194			25.94117	24.21788
	30.28937	30.67708	31.36452	29.30497	26.48047	26.5917			22.64521	22.67681
	28.69027	29.86007	30.81878	30.79701	26.20558	25.42908			22.58932	22.95485
	29.47896	30.3264	31.32155	30.19297	27.16432	26.06626			22.7708	22.70631

内参GAPGH

列1		列2		列3		列4		列5		列6		列7		列8		列9		列10	
平均	20.94542	平均	22.13906	平均	23.36126	平均	22.46933	平均	20.674	平均	20.63236	平均	#DIV/0!	平均	#NUM!	平均	#NUM!	平均	17.87149
标准误差	0.069696	标准误差	0.110874	标准误差	0.06674	标准误差	0.100471	标准误差	0.12357	标准误差	0.068757	标准误差	65535	标准误差	65535	标准误差	0.492651	标准误差	0.31516
中位数	20.95925	中位数	22.18073	中位数	23.3133	中位数	22.4264	中位数	20.5663	中位数	20.7342	中位数	#NUM!	中位数	#NUM!	中位数	17.1455	中位数	17.6522
众数	#N/A	众数	#N/A	众数	#N/A	众数	#N/A	众数	#N/A	众数	#N/A	众数	#N/A	众数	#N/A	众数	#N/A	众数	#N/A
标准差	0.209088	标准差	0.332623	标准差	0.200219	标准差	0.301413	标准差	0.37071	标准差	0.20627	标准差	#DIV/0!	标准差	#DIV/0!	标准差	1.477953	标准差	0.94548
方差	0.043718	方差	0.110638	方差	0.040088	方差	0.09085	方差	0.137426	方差	0.042547	方差	#DIV/0!	方差	#DIV/0!	方差	2.184344	方差	0.893932
峰度	-1.39786	峰度	-1.69217	峰度	-0.9993	峰度	-0.90467	峰度	-0.62459	峰度	0.406634	峰度	#DIV/0!	峰度	#DIV/0!	峰度	-0.10302	峰度	0.086779
偏度	0.012636	偏度	-0.12379	偏度	0.614364	偏度	0.731604	偏度	-0.16468	偏度	-1.1136	偏度	#DIV/0!	偏度	#DIV/0!	偏度	1.034847	偏度	0.152324
区域	0.573192	区域	0.922826	区域	0.5803	区域	0.80628	区域	1.159	区域	0.5891	区域	0	区域	0	区域	4.1766	区域	3.1718
最小值	20.64131	最小值	21.67337	最小值	23.1217	最小值	22.1862	最小值	20.0417	最小值	20.2189	最小值	0	最小值	0	最小值	15.825	最小值	16.3241
最大值	21.2145	最大值	22.5962	最大值	23.702	最大值	22.99248	最大值	21.2007	最大值	20.808	最大值	0	最大值	0	最大值	20.0016	最大值	19.4959
求和	188.5088	求和	199.2515	求和	210.2513	求和	202.224	求和	186.066	求和	185.6912	求和	0	求和	0	求和	157.3437	求和	160.8434
观测数	9	观测数	9	观测数	9	观测数	9	观测数	9	观测数	9	观测数	0	观测数	0	观测数	9	观测数	9
置信度(95	0.160719	置信度(95	0.255677	置信度(95	0.153902	置信度(95	0.231687	置信度(95	0.284953	置信度(95	0.158553	置信度(95	#NUM!	置信度(95	#NUM!	置信度(95	1.136055	置信度(95	0.72676

PPAR

列1		列2		列3		列4		列5		列6		列7		列8		列9		列10	
平均	29.50303	平均	30.2571	平均	31.06567	平均	29.73389	平均	26.74972	平均	25.87347	平均	#DIV/0!	平均	#NUM!	平均	23.83648	平均	23.68202
标准误差	0.166262	标准误差	0.190736	标准误差	0.104542	标准误差	0.169582	标准误差	0.166302	标准误差	0.158242	标准误差	65535	标准误差	65535	标准误差	0.520387	标准误差	0.307312
中位数	29.47896	中位数	30.3264	中位数	31.17968	中位数	29.45895	中位数	26.50026	中位数	26.02194	中位数	#NUM!	中位数	#NUM!	中位数	23.18118	中位数	23.69131
众数	#N/A	众数	#N/A	众数	#N/A	众数	#N/A	众数	#N/A	众数	#N/A	众数	#N/A	众数	#N/A	众数	#N/A	众数	#N/A
标准差	0.498786	标准差	0.572209	标准差	0.313625	标准差	0.508745	标准差	0.498906	标准差	0.474727	标准差	#DIV/0!	标准差	#DIV/0!	标准差	1.561162	标准差	0.921935
方差	0.248788	方差	0.327424	方差	0.09836	方差	0.258822	方差	0.248907	方差	0.225366	方差	#DIV/0!	方差	#DIV/0!	方差	2.437227	方差	0.849964
峰度	-0.20027	峰度	0.862045	峰度	-1.36492	峰度	1.105289	峰度	-1.49459	峰度	-1.17957	峰度	#DIV/0!	峰度	#DIV/0!	峰度	-0.07683	峰度	-1.1631
偏度	-0.24436	偏度	0.378177	偏度	-0.50715	偏度	1.321196	偏度	0.514783	偏度	-0.11608	偏度	#DIV/0!	偏度	#DIV/0!	偏度	1.149238	偏度	0.51177
区域	1.599098	区域	1.986685	区域	0.858778	区域	1.492038	区域	1.323573	区域	1.408455	区域	0	区域	0	区域	4.156025	区域	2.467585
最小值	28.69027	最小值	29.35748	最小值	30.55681	最小值	29.30497	最小值	26.20558	最小值	25.18324	最小值	0	最小值	0	最小值	22.58932	最小值	22.67681
最大值	30.28937	最大值	31.34417	最大值	31.41559	最大值	30.79701	最大值	27.52915	最大值	26.5917	最大值	0	最大值	0	最大值	26.74534	最大值	25.1444
求和	265.5273	求和	272.3139	求和	279.5911	求和	267.605	求和	240.7475	求和	232.8612	求和	0	求和	0	求和	214.5283	求和	213.1382
观测数	9	观测数	9	观测数	9	观测数	9	观测数	9	观测数	9	观测数	0	观测数	0	观测数	9	观测数	9
置信度(95	0.383401	置信度(95	0.439839	置信度(95	0.241073	置信度(95	0.391056	置信度(95	0.383493	置信度(95	0.364908	置信度(95	#NUM!	置信度(95	#NUM!	置信度(95	1.200015	置信度(95	0.708662

ΔCT

9.076522	8.545975	8.22009	7.570112	6.433578	5.822235			6.968218	6.309314
8.114452	7.816656	7.142545	6.73178	5.500205	4.964341			6.04873	5.401174
8.468321	7.975113	7.718141	7.094623	5.968063	4.942596			6.035675	5.706012
9.153453	8.747967	7.97848	7.158424	6.220325	5.708409			6.949141	6.438296
8.144892	7.684108	7.008612	7.032549	6.277711	4.567297			5.845809	5.400314
8.44703	7.974482	8.142157	6.875031	6.328451	5.287736			5.939574	5.675384
9.330113	8.70493	8.004718	7.116574	5.914165	5.783696			6.820215	6.352711
7.916715	7.679342	7.116778	8.601413	5.268478	4.817176			6.217115	5.342446
8.367002	7.933836	8.008253	7.200494	6.770518	5.276558			6.360099	5.669108

ΔΔCT

-0.07693	-0.60748	-0.93336	-1.58334	-2.71988	-3.33122			-2.18524	-2.84414
-0.03044	-0.32824	-1.00235	-1.41311	-2.64469	-3.18055			-2.09616	-2.74372
0.021291	-0.47192	-0.72889	-1.35241	-2.47897	-3.50443			-2.41135	-2.74102
0	-0.40549	-1.17497	-1.99503	-2.93313	-3.44504			-2.20431	-2.71516
0	-0.46078	-1.13628	-1.11234	-1.86718	-3.57759			-2.29908	-2.74458
0	-0.47255	-0.30487	-1.572	-2.11858	-3.15929			-2.50746	-2.77165
0.176659	-0.44852	-1.14874	-2.03688	-3.23929	-3.36976			-2.33324	-2.80074
-0.22818	-0.46555	-1.02811	0.456521	-2.87641	-3.32772			-1.92778	-2.80245
-0.08003	-0.51319	-0.43878	-1.24654	-1.67651	-3.17047			-2.08693	-2.77792

$2^{-ΔΔCT}$

#REF!

	1.05458	1.5214	1.9055	2.9854	6.5458	9.9854			4.5245	7.1325
	1.02125	1.2545	1.9985	2.6542	6.2145	8.9984			4.2545	6.6545
	0.9854	1.3854	1.6545	2.5452	5.5423	11.2546			5.2894	6.6421
	1	1.323262	2.251605	3.967426	7.5847	10.80223			4.58452	6.5245
	1	1.374786	2.192219	2.156269	3.63207	11.838			4.894685	6.658452
	1	1.386004	1.234417	2.962099	4.320894	8.867232			5.65245	6.784125
	0.88512	1.363192	2.211162	4.083793	9.371012	10.2548			5.011545	6.92186
	1.17072	1.379319	2.03439	0.729531	7.2933	9.96127			3.787328	6.930008
	1.056838	1.42547	1.354045	2.365705	3.183857	8.935965			4.227456	6.813599
	1.019323	1.379259	1.870704	2.716625	5.965381	10.09977	#DIV/0!	#DIV/0!	4.69182	6.784627
AVE	1.01878	1.375339	1.87964	2.710804	5.897639	10.10992	#DIV/0!	#DIV/0!	4.692997	6.772091
		p=0		p=0.03		p=0				p=0
	1d	4d	8d	14d						

$2^{-ΔΔCT}$

列1		列2		列3		列4		列5		列6		列7		列8		列9		列10	
平均	1.019323	平均	1.379259	平均	1.870704	平均	2.716625	平均	5.965381	平均	10.09977	平均	#DIV/0!	平均	#NUM!	平均	#NUM!	平均	6.784627
标准误差	0.025237	标准误差	0.02398	标准误差	0.125125	标准误差	0.333023	标准误差	0.671311	标准误差	0.353385	标准误差	65535	标准误差	65535	标准误差	0.193211	标准误差	0.062656
中位数	1	中位数	1.379319	中位数	1.9985	中位数	2.6542	中位数	6.2145	中位数	9.9854	中位数	#NUM!	中位数	#NUM!	中位数	4.58452	中位数	6.784125
众数	1	众数	#N/A	众数	#N/A	众数	#N/A	众数	#N/A	众数	#N/A	众数	#N/A	众数	#N/A	众数	#N/A	众数	#N/A
标准差	0.07571	标准差	0.071939	标准差	0.375374	标准差	0.99907	标准差	2.013932	标准差	1.060155	标准差	#DIV/0!	标准差	#DIV/0!	标准差	0.579632	标准差	0.187968
方差	0.005732	方差	0.005175	方差	0.140906	方差	0.99814	方差	4.055923	方差	1.123929	方差	#DIV/0!	方差	#DIV/0!	方差	0.335973	方差	0.035332
峰度	2.398938	峰度	2.078695	峰度	-0.74658	峰度	1.250888	峰度	-0.63968	峰度	-1.01629	峰度	#DIV/0!	峰度	#DIV/0!	峰度	-0.4317	峰度	-0.07015
偏度	0.403303	偏度	0.366648	偏度	-0.8239	偏度	-0.56795	偏度	0.148582	偏度	0.317464	偏度	#DIV/0!	偏度	#DIV/0!	偏度	0.174335	偏度	0.558281
区域	0.2856	区域	0.2669	区域	1.017188	区域	3.354262	区域	6.187155	区域	2.970768	区域	0	区域	0	区域	1.865122	区域	0.608
最小值	0.88512	最小值	1.2545	最小值	1.234417	最小值	0.729531	最小值	3.183857	最小值	8.867232	最小值	0	最小值	0	最小值	3.787328	最小值	6.5245
最大值	1.17072	最大值	1.5214	最大值	2.251605	最大值	4.083793	最大值	9.371012	最大值	11.838	最大值	0	最大值	0	最大值	5.65245	最大值	7.1325
求和	9.173908	求和	12.41333	求和	16.83634	求和	24.44962	求和	53.68843	求和	90.8979	求和	0	求和	0	求和	42.22638	求和	61.06164
观测数	9	观测数	9	观测数	9	观测数	9	观测数	9	观测数	9	观测数	0	观测数	0	观测数	9	观测数	9
置信度(95	0.058196	置信度(95	0.055297	置信度(95	0.288538	置信度(95	0.767953	置信度(95	1.548045	置信度(95	0.814908	置信度(95	#NUM!	置信度(95	#NUM!	置信度(95	0.445545	置信度(95	0.144485
0.961127	0.961127	1.323962	1.434556	1.582166	2.159242	1.948672	3.484578	4.417336	7.513427	9.284859	10.91467					#NUM!	#NUM!	6.640142	6.929112

0.058196	0.058196	0.055297	0.055297	0.288538	0.288538	0.767953	0.767953	1.548045	1.548045	0.814908	0.814908					0.445545	0.445545	0.144485	0.144485

	c1d	v1d	c4d	v4d	c8d	v8d	c14d	v14d	c14d	v14d
内参GAPGH										
	20.8545	21.5455	23.3955	22.4522	20.4596	20.4511			17.5422	17.4877
	21.2144	22.4218	23.3677	22.6951	20.8561	20.6119			17.4955	17.6522
	21.0025	22.5962	23.2419	22.2655	20.5322	20.895			17.1455	17.9853
	20.64131	21.67337	23.302	22.0862	20.8938	20.4936			19.7962	18.7061
	20.73682	21.77366	23.3133	22.1884	21.0785	20.7888			16.2548	19.4959
	21.32907	22.39552	23.1217	22.8221	21.2007	20.7342			20.0016	18.7546
	20.95925	22.00721	23.6598	22.4264	20.5663	20.898			15.825	16.3241
	20.74355	21.78073	23.2012	22.1956	20.0371	20.2189			16.1722	16.6124
	21.61196	22.69256	23.5482	23.1248	19.8417	20.7897			16.4107	16.3372
CEBP										
	26.80025	26.78074	29.08938	26.89204	24.85671	24.04342			21.98506	21.12582
	26.45894	27.12434	28.54468	26.67716	24.48183	23.47439			20.94595	20.86802
	26.52954	27.4139	28.6021	26.62343	24.08916	23.81436			20.89475	21.2742
	26.60511	26.6298	29.09146	26.38409	25.5128	23.61146			23.64793	22.44439
	26.07282	26.6618	28.445	26.41023	24.48628	23.59289			20.05523	22.54755
	26.90247	27.07377	28.15006	26.81318	24.9016	24.00462			23.37034	22.2318
	26.87725	27.07178	29.43606	26.81866	24.58023	24.1618			20.26037	20.23485
	25.90846	26.39588	28.33681	26.13039	23.7442	23.03215			19.97996	20.0786
	27.10892	28.24002	28.89181	27.99617	23.61236	24.71376			20.67306	19.10191

内参GAPGH

列1		列2		列3		列4		列5		列6		列7		列8		列9		列10	
平均	18.06242	平均	7.395944	平均	19.60665	平均	7.490311	平均	20.60733	平均	20.65347	平均	#DIV/0!	平均	#NUM!	平均	#NUM!	平均	17.70617
标准误差	0.741378	标准误差	3.699163	标准误差	0.932579	标准误差	3.745328	标准误差	0.151774	标准误差	0.07625	标准误差	65535	标准误差	65535	标准误差	0.512108	标准误差	0.380265
中位数	16.6528	中位数	0	中位数	17.8185	中位数	0	中位数	20.5663	中位数	20.7342	中位数	#NUM!	中位数	#NUM!	中位数	17.1455	中位数	17.6522
众数	#N/A	众数	0	众数	#N/A	众数	0	众数	#N/A	众数	#N/A	众数	#N/A	众数	#N/A	众数	#N/A	众数	#N/A
标准差	2.224134	标准差	11.09749	标准差	2.797736	标准差	11.23598	标准差	0.455322	标准差	0.22875	标准差	#DIV/0!	标准差	#DIV/0!	标准差	1.536323	标准差	1.140794
方差	4.946773	方差	123.1542	方差	7.827324	方差	126.2473	方差	0.207318	方差	0.052327	方差	#DIV/0!	方差	#DIV/0!	方差	2.360289	方差	1.30141
峰度	-1.68569	峰度	-1.70345	峰度	-1.71063	峰度	-1.71271	峰度	-0.62233	峰度	-0.10091	峰度	#DIV/0!	峰度	#DIV/0!	峰度	-0.27106	峰度	-1.23943
偏度	0.859167	偏度	0.859603	偏度	0.854797	偏度	0.857498	偏度	-0.48613	偏度	-0.80917	偏度	#DIV/0!	偏度	#DIV/0!	偏度	1.012776	偏度	0.149631
区域	4.7731	区域	22.5962	区域	5.803309	区域	22.6951	区域	1.359	区域	0.6791	区域	0	区域	0	区域	4.1766	区域	3.1718
最小值	16.4413	最小值	0	最小值	17.59219	最小值	0	最小值	19.8417	最小值	20.2189	最小值	0	最小值	0	最小值	15.825	最小值	16.3241
最大值	21.2144	最大值	22.5962	最大值	23.3955	最大值	22.6951	最大值	21.2007	最大值	20.898	最大值	0	最大值	0	最大值	20.0016	最大值	19.4959
求和	162.5618	求和	66.5635	求和	176.4598	求和	67.4128	求和	185.466	求和	185.8812	求和	0	求和	0	求和	156.6437	求和	159.3555
观测数	9	观测数	9	观测数	9	观测数	9	观测数	9	观测数	9	观测数	0	观测数	0	观测数	9	观测数	9
置信度(95	1.709621	置信度(95	8.530285	置信度(95	2.15053	置信度(95	8.636741	置信度(95	0.349991	置信度(95	0.175833	置信度(95	#NUM!	置信度(95	#NUM!	置信度(95	1.180923	置信度(95	0.876892
16.3528	19.77204	-1.13434	15.92623	17.45612	21.75718	-1.14643	16.12705	20.25734	20.95732	20.47763	20.8293					#NUM!	#NUM!	16.82927	18.58306

CEBP

列1		列2		列3		列4		列5		列6		列7		列8		列9		列10	
平均	26.58486	平均	27.04356	平均	28.73193	平均	26.74948	平均	24.47391	平均	23.82765	平均	#DIV/0!	平均	#NUM!	平均	21.31252	平均	21.10079
标准误差	0.131573	标准误差	0.181904	标准误差	0.139807	标准误差	0.176242	标准误差	0.198712	标准误差	0.159616	标准误差	65535	标准误差	65535	标准误差	0.461315	标准误差	0.391803
中位数	26.60511	中位数	27.07178	中位数	28.6021	中位数	26.67716	中位数	24.48628	中位数	23.81436	中位数	#NUM!	中位数	#NUM!	中位数	20.89475	中位数	21.12582
众数	#N/A	众数	#N/A	众数	#N/A	众数	#N/A	众数	#N/A	众数	#N/A	众数	#N/A	众数	#N/A	众数	#N/A	众数	#N/A
标准差	0.39472	标准差	0.545712	标准差	0.419422	标准差	0.528725	标准差	0.596137	标准差	0.478848	标准差	#DIV/0!	标准差	#DIV/0!	标准差	1.383945	标准差	1.175409
方差	0.155804	方差	0.297801	方差	0.175915	方差	0.27955	方差	0.35538	方差	0.229295	方差	#DIV/0!	方差	#DIV/0!	方差	1.915303	方差	1.381586
峰度	-0.45128	峰度	2.373455	峰度	-0.87725	峰度	4.320847	峰度	-0.16775	峰度	0.795228	峰度	#DIV/0!	峰度	#DIV/0!	峰度	-0.55707	峰度	-0.79679
偏度	-0.61392	偏度	1.332896	偏度	0.336025	偏度	1.75457	偏度	0.130335	偏度	0.260717	偏度	#DIV/0!	偏度	#DIV/0!	偏度	0.961248	偏度	-0.27818
区域	1.200466	区域	1.844139	区域	1.286	区域	1.865785	区域	1.90044	区域	1.681608	区域	0	区域	0	区域	3.667973	区域	3.445641
最小值	25.90846	最小值	26.39588	最小值	28.15006	最小值	26.13039	最小值	23.61236	最小值	23.03215	最小值	0	最小值	0	最小值	19.97996	最小值	19.10191
最大值	27.10892	最大值	28.24002	最大值	29.43606	最大值	27.99617	最大值	25.5128	最大值	24.71376	最大值	0	最大值	0	最大值	23.64793	最大值	22.54755
求和	239.2638	求和	243.392	求和	258.5874	求和	240.7454	求和	220.2652	求和	214.4488	求和	0	求和	0	求和	191.8126	求和	189.9071
观测数	9	观测数	9	观测数	9	观测数	9	观测数	9	观测数	9	观测数	0	观测数	0	观测数	9	观测数	9
置信度(95	0.303409	置信度(95	0.419471	置信度(95	0.322397	置信度(95	0.406414	置信度(95	0.458232	置信度(95	0.368075	置信度(95	#NUM!	置信度(95	#NUM!	置信度(95	1.063794	置信度(95	0.903499

ΔCT

5.945752	5.235239	5.693876	4.439838	4.397111	3.592322			4.442863	3.638117
5.244543	4.702541	5.176982	3.982061	3.625734	2.862488			3.450447	3.215816
5.527037	4.817704	5.360205	4.357931	3.556959	2.919358			3.749253	3.288902
5.9638	4.956423	5.789457	4.297892	4.619001	3.117862			3.85173	3.738294
5.336	4.888138	5.131703	4.221835	3.407777	2.80409			3.800425	3.051651
5.5734	4.678247	5.028356	3.991082	3.700895	3.27042			3.368739	3.477197
5.917998	5.064563	5.776256	4.392257	4.013934	3.2638			4.43537	3.910747
5.164906	4.615154	5.135612	3.934788	3.707104	2.813248			3.807758	3.466203
5.496961	5.547461	5.34361	4.871373	3.770661	3.924057			4.262359	2.76471

ΔΔCT

-0.01805	-0.72856	-0.26992	-1.52396	-1.56669	-2.37148			-1.52094	-2.32568
-0.09146	-0.63346	-0.15902	-1.35394	-1.71027	-2.47351			-1.88555	-2.12018
-0.04636	-0.7557	-0.2132	-1.21547	-2.01644	-2.65404			-1.82415	-2.2845
0	-1.00738	-0.17434	-1.66591	-1.3448	-2.84594			-2.11207	-2.22551
0	-0.44786	-0.2043	-1.11417	-1.92822	-2.53191			-1.53557	-2.28435
0	-0.89515	-0.54504	-1.58232	-1.8725	-2.30298			-2.20466	-2.0962
-0.0458	-0.89924	-0.18754	-1.57154	-1.94987	-2.7			-1.52843	-2.05305
-0.17109	-0.72085	-0.20039	-1.40121	-1.6289	-2.52275			-1.52824	-1.8698
-0.07644	-0.02594	-0.22979	-0.70203	-1.80274	-1.64934			-1.31104	-2.80869

$2^{-ΔΔCT}$

1.012545	1.654125	1.204973	2.865422	2.951255	5.145688			2.85944	4.985455
1.065214	1.548951	1.116106	2.547896	3.258966	5.521455			3.67846	4.325688
1.032545	1.685422	1.158667	2.315478	4.026544	6.254789			3.52569	4.845622
1	2.005456	1.127984	3.160621	2.53186	7.141366			4.30151	4.652131
1	1.36257	1.151567	2.158985	3.788496	5.748744			2.8885	4.845122
1	1.855863	1.45718	2.98329	3.645452	4.90788			4.585621	4.254622
1.032145	1.861106	1.138317	2.961167	3.845562	6.456541			2.87428	4.129676
1.125455	1.645333	1.148461	2.632472	3.080837	5.71249			2.873907	3.638639
1.054221	1.018079	1.172025	1.624083	3.473935	3.124662			2.473503	6.959964

$2^{-ΔΔCT}$

列1		列2		列3		列4		列5		列6		列7		列8		列9		列10	
平均	1.035792	平均	1.626323	平均	1.186142	平均	2.583268	平均	2.916913	平均	5.557068	平均	#DIV/0!	平均	#NUM!	平均	#NUM!	平均	4.737435
标准误差	0.01377	标准误差	0.09872	标准误差	0.034952	标准误差	0.16207	标准误差	0.223829	标准误差	0.379607	标准误差	65535	标准误差	65535	标准误差	0.242823	标准误差	0.312052
中位数	1.032145	中位数	1.654125	中位数	1.151567	中位数	2.632472	中位数	3.080837	中位数	5.71249	中位数	#NUM!	中位数	#NUM!	中位数	3.788496	中位数	4.652131
众数	1	众数	#N/A	众数	#N/A	众数	#N/A	众数	#N/A	众数	#N/A	众数	#N/A	众数	#N/A	众数	#N/A	众数	#N/A
标准差	0.04131	标准差	0.296161	标准差	0.104857	标准差	0.486211	标准差	0.671488	标准差	1.138821	标准差	#DIV/0!	标准差	#DIV/0!	标准差	0.728469	标准差	0.936155
方差	0.001707	方差	0.087712	方差	0.010995	方差	0.236401	方差	0.450896	方差	1.296914	方差	#DIV/0!	方差	#DIV/0!	方差	0.530667	方差	0.876387
峰度	1.932587	峰度	1.28598	峰度	7.485277	峰度	0.449077	峰度	-1.19626	峰度	2.181756	峰度	#DIV/0!	峰度	#DIV/0!	峰度	-0.73902	峰度	4.524371
偏度	1.373268	偏度	-1.01507	偏度	2.669419	偏度	-0.902	偏度	-0.27614	偏度	-1.05334	偏度	#DIV/0!	偏度	#DIV/0!	偏度	0.752479	偏度	1.814356
区域	0.125455	区域	0.987376	区域	0.341074	区域	1.536538	区域	1.907756	区域	4.016704	区域	0	区域	0	区域	2.112118	区域	3.321325
最小值	1	最小值	1.018079	最小值	1.116106	最小值	1.624083	最小值	1.937806	最小值	3.124662	最小值	0	最小值	0	最小值	3.373503	最小值	3.638639
最大值	1.125455	最大值	2.005456	最大值	1.45718	最大值	3.160621	最大值	3.845562	最大值	7.141366	最大值	0	最大值	0	最大值	5.485621	最大值	6.959964
求和	9.322125	求和	14.6369	求和	10.67528	求和	23.24941	求和	26.25222	求和	50.01361	求和	0	求和	0	求和	38.16041	求和	42.63692
观测数	9	观测数	9	观测数	9	观测数	9	观测数	9	观测数	9	观测数	0	观测数	0	观测数	9	观测数	9

**AVE**	1.035792	1.626323	1.186142	2.583268	3.400323	5.557068	#DIV/0!	#DIV/0!	3.340101	4.737435
3	1.035792	1.626323	1.186142	2.583268	3.400323	5.557068			3.340101	4.737435
		p=0		p=0		p=0				p=.003
	1d	4d	8d	14d						

置信度(95	0.031754	置信度(95	0.22765	置信度(95	0.0806	置信度(95	0.373735	置信度(95	0.516151	置信度(95	0.875375	置信度(95	#NUM!	置信度(95	#NUM!	置信度(95	0.559951	置信度(95	0.719593
1.004038	1.853973	1.398673	1.853973	1.105542	1.266743	2.209533	2.957003	2.400762	3.433064	4.681693	6.432444					#NUM!	#NUM!	4.017843	5.457028

	c1d	v1d	c4d	v4d	c8d	v8d	c14d	v14d	c14d	v14d
**内参GAPGH**										
	20.8545	21.5455	23.3955	22.4522	20.4596	20.4511			17.5422	17.4877
	21.2144	22.4218	23.3677	22.6951	20.8561	20.6119			17.4955	17.6522
	21.0025	22.5962	23.2419	22.2655	20.5322	20.895			17.1455	17.9853
	20.64131	21.67337	23.302	22.0862	20.8938	20.4936			19.7962	18.7061
	20.73682	21.77366	23.3133	22.1884	21.0785	20.7888			16.2548	19.4959
	21.32907	22.39552	23.1217	22.8221	21.2007	20.7342			20.0016	18.7546
	20.95925	22.00721	23.6598	22.4264	20.5663	20.898			15.825	16.3241
	20.74355	21.78073	23.2012	22.1956	20.0371	20.2189			16.1722	16.6124
	21.61196	22.69256	23.5482	23.1248	19.8417	20.7897			16.4107	16.3372
**ADI**										
	25.10946	25.87546	26.86169	25.64122	23.8153	22.89181			20.27607	19.65253
	25.78448	27.05012	27.18296	26.09622	24.48505	23.38528			20.56747	20.1699
	25.34445	26.89305	26.65018	25.36846	23.88796	23.38572			19.91689	20.15864
	25.00156	26.03982	26.81819	25.59853	24.24284	23.32768			22.26638	20.61301
	25.43883	26.49891	27.18788	25.25547	24.42928	23.04122			19.85154	21.74435
	25.72997	26.8063	26.68881	26.06072	23.68785	22.80493			23.21595	20.6409
	25.15352	26.19612	26.98539	25.76965	23.96812	23.0381			18.08842	18.64304
	25.48486	26.54826	27.12216	25.65026	24.25243	24.12307			19.21092	19.07309
	25.71687	26.78866	26.78506	26.06229	24.8575	23.65013			19.22972	19.86092

内参GAPGH	列1		列2		列3		列4		列5		列6		列7		列8		列9		列10	
	平均	21.01037	平均	22.09851	平均	23.35014	平均	22.47292	平均	20.60733	平均	20.65347	平均	#DIV/0!	平均	#NUM!	平均	#NUM!	平均	17.70617
	标准误差	0.106348	标准误差	0.144084	标准误差	0.056179	标准误差	0.114823	标准误差	0.151774	标准误差	0.07625	标准误差	65535	标准误差	65535	标准误差	0.512108	标准误差	0.380265
	中位数	20.95925	中位数	22.00721	中位数	23.3133	中位数	22.4264	中位数	20.5663	中位数	20.7342	中位数	#NUM!	中位数	#NUM!	中位数	17.1455	中位数	17.6522
	众数	#N/A	众数	#N/A	众数	#N/A	众数	#N/A	众数	#N/A	众数	#N/A	众数	#N/A	众数	#N/A	众数	#N/A	众数	#N/A
	标准差	0.319043	标准差	0.432251	标准差	0.168538	标准差	0.344468	标准差	0.455322	标准差	0.22875	标准差	#DIV/0!	标准差	#DIV/0!	标准差	1.536323	标准差	1.140794
	方差	0.101788	方差	0.186841	方差	0.028405	方差	0.118658	方差	0.207318	方差	0.052327	方差	#DIV/0!	方差	#DIV/0!	方差	2.360289	方差	1.30141
	峰度	-0.1029	峰度	-1.8691	峰度	0.108571	峰度	-0.10889	峰度	-0.62233	峰度	-0.10091	峰度	#DIV/0!	峰度	#DIV/0!	峰度	-0.27106	峰度	-1.23943
	偏度	0.816191	偏度	0.15885	偏度	0.701742	偏度	0.862364	偏度	-0.48613	偏度	-0.80917	偏度	#DIV/0!	偏度	#DIV/0!	偏度	1.012776	偏度	0.149631
	区域	0.970654	区域	1.147061	区域	0.5381	区域	1.0386	区域	1.359	区域	0.6791	区域	0	区域	0	区域	4.1766	区域	3.1718
	最小值	20.64131	最小值	21.5455	最小值	23.1217	最小值	22.0862	最小值	19.8417	最小值	20.2189	最小值	0	最小值	0	最小值	15.825	最小值	16.3241
	最大值	21.61196	最大值	22.69256	最大值	23.6598	最大值	23.1248	最大值	21.2007	最大值	20.898	最大值	0	最大值	0	最大值	20.0016	最大值	19.4959
	求和	189.0934	求和	198.8866	求和	210.1513	求和	202.2563	求和	185.466	求和	185.8812	求和	0	求和	0	求和	156.6437	求和	159.3555
	观测数	9	观测数	9	观测数	9	观测数	9	观测数	9	观测数	9	观测数	0	观测数	0	观测数	9	观测数	9
	置信度(95	0.245238	置信度(95	0.332258	置信度(95	0.12955	置信度(95	0.264781	置信度(95	0.349991	置信度(95	0.175833	置信度(95	#NUM!	置信度(95	#NUM!	置信度(95	1.180923	置信度(95	0.876892

ADI	列1		列2		列3		列4		列5		列6		列7		列8		列9		列10	
	平均	25.41822	平均	26.52186	平均	26.92026	平均	25.72254	平均	24.1807	平均	23.29422	平均	#DIV/0!	平均	#NUM!	平均	20.29149	平均	20.06182
	标准误差	0.096454	标准误差	0.135732	标准误差	0.06915	标准误差	0.101543	标准误差	0.12521	标准误差	0.137558	标准误差	65535	标准误差	65535	标准误差	0.527197	标准误差	0.304811
	中位数	25.43883	中位数	26.54826	中位数	26.86169	中位数	25.65026	中位数	24.24284	中位数	23.32768	中位数	#NUM!	中位数	#NUM!	中位数	19.91689	中位数	20.15864
	众数	#N/A	众数	#N/A	众数	#N/A	众数	#N/A	众数	#N/A	众数	#N/A	众数	#N/A	众数	#N/A	众数	#N/A	众数	#N/A
	标准差	0.289361	标准差	0.407195	标准差	0.207449	标准差	0.304629	标准差	0.375631	标准差	0.412674	标准差	#DIV/0!	标准差	#DIV/0!	标准差	1.581592	标准差	0.914434
	方差	0.08373	方差	0.165808	方差	0.043035	方差	0.092799	方差	0.141099	方差	0.1703	方差	#DIV/0!	方差	#DIV/0!	方差	2.501434	方差	0.836189
	峰度	-1.53082	峰度	-1.17789	峰度	-1.64743	峰度	-1.16484	峰度	-0.4212	峰度	0.7862	峰度	#DIV/0!	峰度	#DIV/0!	峰度	0.33483	峰度	0.506869
	偏度	-0.09518	偏度	-0.41911	偏度	0.179474	偏度	-0.10755	偏度	0.45779	偏度	0.916506	偏度	#DIV/0!	偏度	#DIV/0!	偏度	0.789962	偏度	0.258054
	区域	0.782922	区域	1.174661	区域	0.537702	区域	0.840746	区域	1.169652	区域	1.318143	区域	0	区域	0	区域	5.12753	区域	3.101315
	最小值	25.00156	最小值	25.87546	最小值	26.65018	最小值	25.25547	最小值	23.68785	最小值	22.80493	最小值	0	最小值	0	最小值	18.08842	最小值	18.64304
	最大值	25.78448	最大值	27.05012	最大值	27.18788	最大值	26.09622	最大值	24.8575	最大值	24.12307	最大值	0	最大值	0	最大值	23.21595	最大值	21.74435
	求和	228.764	求和	238.6967	求和	242.2823	求和	231.5028	求和	217.6263	求和	209.6479	求和	0	求和	0	求和	182.6234	求和	180.5564
	观测数	9	观测数	9	观测数	9	观测数	9	观测数	9	观测数	9	观测数	0	观测数	0	观测数	9	观测数	9
	置信度(95	0.222422	置信度(95	0.312998	置信度(95	0.159459	置信度(95	0.234158	置信度(95	0.288736	置信度(95	0.317209	置信度(95	#NUM!	置信度(95	#NUM!	置信度(95	1.215719	置信度(95	0.702896

ΔCT

4.254959	4.329964	3.466194	3.189016	3.355698	2.440708			2.73387	2.164827
4.57008	4.628325	3.815263	3.40112	3.628946	2.773381			3.071974	2.517698
4.341949	4.296854	3.408277	3.102964	3.355763	2.490721			2.771387	2.173338
4.360249	4.366447	3.516192	3.512335	3.34904	2.834078			2.470183	1.906906
4.702005	4.725246	3.874579	3.067074	3.350777	2.252419			3.596737	2.248451
4.400901	4.410783	3.567107	3.238619	2.487152	2.070727			3.214354	1.886296
4.194266	4.1889	3.32559	3.343252	3.401821	2.140104			2.263425	2.318937
4.741306	4.767534	3.920958	3.454656	4.215335	3.90417			3.03872	2.460691
4.104904	4.096095	3.236857	2.937493	5.015804	2.860429			2.819018	3.523721

ΔΔCT

-0.10529	-0.03029	-0.89406	-1.17123	-1.00455	-1.91954			-1.62638	-2.19542
-0.13192	-0.07368	-0.88674	-1.30089	-1.07306	-1.92862			-1.63003	-2.18431
-0.05895	-0.10405	-0.99262	-1.29794	-1.04514	-1.91018			-1.62951	-2.22756
0	0.006197	-0.84406	-0.84791	-1.01121	-1.52617			-1.89007	-2.45334
0	0.023241	-0.82743	-1.63493	-1.35123	-2.44959			-1.10527	-2.45355
0	0.009882	-0.83379	-1.16228	-1.91375	-2.33017			-1.18655	-2.51461
-0.16598	-0.17135	-1.03466	-1.017	-0.95843	-2.22015			-2.09682	-2.04131
0.039301	0.065529	-0.78105	-1.24735	-0.48667	-0.79784			-1.66328	-2.24131
-0.296	-0.30481	-1.16404	-1.46341	0.614903	-1.54047			-1.58188	-0.87718

$2^{-\Delta\Delta CT}$

	1.075442	1.021141	1.854456	2.245795	2.001545	3.765845			3.075486	4.556451
	1.095412	1.052214	1.845112	2.456211	2.098542	3.789546			3.083254	4.521599
	1.041563	1.074519	1.985122	2.451214	2.058456	3.741571			3.082154	4.658744
	1	0.995728	1.791502	1.796281	2.010772	2.869798			3.689946	5.445065
	1	0.984074	1.771038	3.093708	2.54313	5.430952			2.145756	5.445856
	1	0.993197	1.778846	2.231951	3.750808	5.000947			2.269677	5.680435
	1.121489	1.125654	2.043609	2.018827	1.93878	4.634936			4.256448	4.096319
	0.973217	0.955743	1.715198	2.367035	1.399591	1.735204			3.154899	4.703209
	1.226872	1.23436	2.234669	2.748037	0.653927	2.898289			2.982394	1.832965
**AVE**	1.053596	1.048126	1.889144	2.375973	2.049501	3.761688	#DIV/0!	#DIV/0!	3.083187	4.533975
3	1.059333	1.048514	1.891061	2.378784	2.050617	3.76301	#DIV/0!	#DIV/0!	3.082224	4.54896
		p=.788		p=.003		p=.002				p=.004
	1d	4d	8d	14d						

$2^{-\Delta\Delta CT}$	列1		列2		列3		列4		列5		列6		列7		列8		列9		列10	
	平均	1.059333	平均	1.048514	平均	1.891061	平均	2.378784	平均	2.050617	平均	3.76301	平均	#DIV/0!	平均	#NUM!	平均	#NUM!	平均	4.54896
	标准误差	0.026819	标准误差	0.028996	标准误差	0.055462	标准误差	0.12731	标准误差	0.277054	标准误差	0.386104	标准误差	65535	标准误差	65535	标准误差	0.214108	标准误差	0.381987
	中位数	1.041563	中位数	1.021141	中位数	1.845112	中位数	2.367035	中位数	2.010772	中位数	3.765845	中位数	#NUM!	中位数	#NUM!	中位数	3.082154	中位数	4.658744
	众数	1	众数	#N/A	众数	#N/A	众数	#N/A	众数	#N/A	众数	#N/A	众数	#N/A	众数	#N/A	众数	#N/A	众数	#N/A
	标准差	0.080457	标准差	0.086987	标准差	0.166386	标准差	0.381931	标准差	0.831163	标准差	1.158313	标准差	#DIV/0!	标准差	#DIV/0!	标准差	0.642323	标准差	1.145962
	方差	0.006473	方差	0.007567	方差	0.027684	方差	0.145871	方差	0.690832	方差	1.341689	方差	#DIV/0!	方差	#DIV/0!	方差	0.412578	方差	1.313228
	峰度	1.161348	峰度	1.687127	峰度	1.015774	峰度	0.622369	峰度	2.388042	峰度	-0.29043	峰度	#DIV/0!	峰度	#DIV/0!	峰度	0.531098	峰度	4.342027
	偏度	1.148866	偏度	1.34113	偏度	1.229418	偏度	0.481146	偏度	0.568788	偏度	-0.25825	偏度	#DIV/0!	偏度	#DIV/0!	偏度	0.294209	偏度	-1.84038
	区域	0.253654	区域	0.278617	区域	0.519471	区域	1.297427	区域	3.096881	区域	3.695748	区域	0	区域	0	区域	2.110692	区域	3.84747
	最小值	0.973217	最小值	0.955743	最小值	1.715198	最小值	1.796281	最小值	0.653927	最小值	1.735204	最小值	0	最小值	0	最小值	2.145756	最小值	1.832965
	最大值	1.226872	最大值	1.23436	最大值	2.234669	最大值	3.093708	最大值	3.750808	最大值	5.430952	最大值	0	最大值	0	最大值	4.256448	最大值	5.680435
	求和	9.533995	求和	9.43663	求和	17.01955	求和	21.40906	求和	18.45555	求和	33.86709	求和	0	求和	0	求和	27.74001	求和	40.94064
	观测数	9	观测数	9	观测数	9	观测数	9	观测数	9	观测数	9	观测数	0	观测数	0	观测数	9	观测数	9
	置信度(95	0.061845	置信度(95	0.066864	置信度(95	0.127895	置信度(95	0.293578	置信度(95	0.638889	置信度(95	0.890358	置信度(95	#NUM!	置信度(95	#NUM!	置信度(95	0.493733	置信度(95	0.880864
	0.997488	1.115378	0.981651	1.115378	1.763166	2.018957	2.085207	2.672362	1.411728	2.689505	2.872652	4.653368					#NUM!	#NUM!	3.668096	5.429824

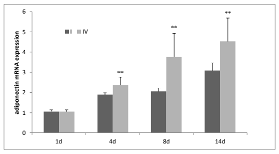

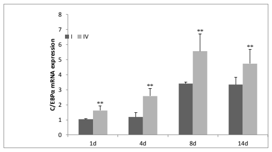

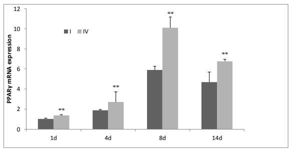

area	grey(average)	PPAR	relative	total					STD		P
297	141.4983	C	98.59207	29281.84		0.717376	control	vibration	0.010898	0.054025	0
306	140.2549		97.34865	29788.69	C/ACTIN C	0.709985	0.707761	1.237513	C	V	
279	145.4373		102.531	28606.16		0.695924					
444	161.0608	V	118.1546	52460.63							
481	155.5821		112.6759	54197.09							
532	148.5508		105.6445	56202.88							
416	141.0264	ACTIN C	98.12019	40818							
510	125.1745		82.26826	41956.81							
462	131.8788		88.97254	41105.31							
544	124.4504	ACTIN V	81.54412	44360	V/ACTIN V	1.182611					
476	134.7794		91.87316	43731.63		1.239311	1.237513				
462	137.1645		94.25825	43547.31		1.290616					
32	42.90625	BACKGROUND									
	42.90625										
	42.90625										
	42.90625										
	42.90625										
	42.90625										
	42.90625										
	42.90625										
	42.90625										
	42.90625										
	42.90625										
	42.90625										

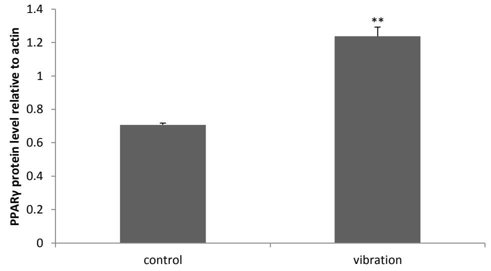

corrected

area	GREY V(AVERAGE)	PPAR	RELATIVE	TOTAL					standard dev.		P
297	141.4983	control	98.59207	29281.84		0.499119	control	vibration	0.09821	0.0958	0.002
306	140.2549		140.2549	42918	C/ACTIN C	0.672285	0.612462	1.156618	C	V	
279	145.4373		145.4373	40577		0.665983					
444	161.0608	vibration	161.0608	71511							
481	155.5821		155.5821	74835							
532	148.5508		148.5508	79029							
416	141.0264	ACTIN C	141.0264	58667							
510	125.1745		125.1745	63839							
462	131.8788		131.8788	60928							
544	124.4504	ACTIN V	124.4504	67701	V/ACTIN V	1.056277					
476	134.7794		134.7794	64155		1.166472	1.156618				
462	137.1645		137.1645	63370		1.247104					
32	42.90625	BACKGROUND									

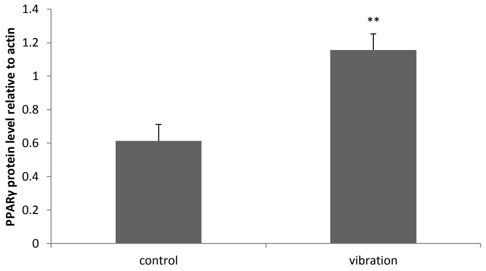

original

	1d		4d		8d	
pp38	C	V	C	V	C	V
	78455	231420	115500	431730	60984	143724
	81697	231608	121326	435968	58174	147132
	80400	227304	119508	428461	63080	137704
p38	295776	258750	344007	343882	162036	148148
	302652	252890	342504	348697	162690	148915
	291648	256564	344540	332408	159408	147744
pp38/p38	0.265251	0.894377	0.335749	1.25546	0.376361	0.970138
	0.269937	0.915845	0.354232	1.250277	0.357576	0.988027
	0.275675	0.885954	0.346862	1.288961	0.395714	0.932045
AVER	0.270288	0.898725	0.345615	1.264899	0.37655	0.963403
STD	0.005221	0.015412	0.009305	0.020999	0.01907	0.028592
	1	3.371808	1.278691	4.679825	1.393146	3.564361
	1.017665	3.452743	1.335459	4.713556	1.348064	3.724869
	1.039296	3.340055	1.307674	4.859394	1.491846	3.513816
CALI. STD	0.019681	0.058105	0.028386	0.095439	0.073538	0.110198
p		0		0		0
CALI. AVER	1.018987	3.388202	1.307275	4.750925	1.411018	3.601015

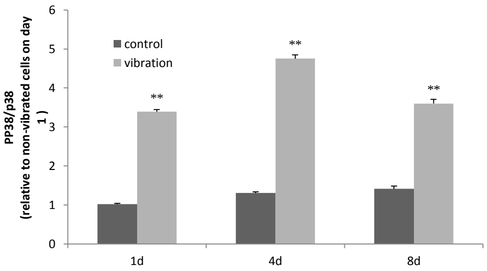

V+SB20358	74039	125048	0.592085	0.528672		control	control+SB	vibration	vibration+SB
	64146	125048	0.512971	52.8672	0.057202	p=0.044		p=0.045	
	60143	125048	0.480959						
C+SB20358	62990	125048	0.503727	0.451344					
	55998	125048	0.447812	45.1344	0.050709				
	50331	125048	0.402493						
V	85624	125048	0.684729	0.644806					
	80678	125048	0.645176	64.4806	0.04011				
	75593	125048	0.604512						
C	71272	125048	0.569957	0.551687					
	71272	125048	0.569957	55.1687	0.316451				
	64418	125048	0.515146						
					standard dev.				
	55.1687	45.1344	64.4806	52.8672					

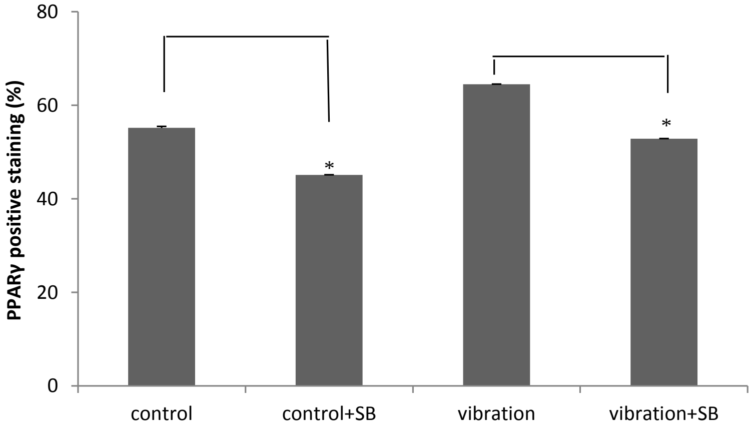

Supplement: S2 File — (PDF) [file pone.0172954.s002.pdf]
